# Disseminating, implementing, and evaluating patient-centered outcomes to improve cardiovascular care using a stepped-wedge design: healthy hearts for Oklahoma

**DOI:** 10.1186/s12913-018-3189-4

**Published:** 2018-06-04

**Authors:** Ann F. Chou, Juell B. Homco, Zsolt Nagykaldi, James W. Mold, F. Daniel Duffy, Steven Crawford, Julie A. Stoner

**Affiliations:** 1 College of Medicine, Department of Family and Preventive Medicine, The University of Oklahoma Health Sciences Center, 900 NE 10th St, Oklahoma City, OK 73104 USA; 2 School of Community Medicine, The University of Oklahoma Health Sciences Center, 4502 E 41st St, Tulsa, OK 74135 USA; 3 College of Public Health, The University of Oklahoma Health Sciences Center, 801 NE 13th St, Oklahoma City, OK 73104 USA

**Keywords:** Primary care, Quality improvement, Practice facilitation, Cardiovascular disease, Patient-centered outcomes, Implementation and dissemination

## Abstract

**Background:**

Cardiovascular disease (CVD) is the leading cause of death in the US and incurs high health care costs. While many initiatives promote the implementation of ABCS (aspirin therapy, blood pressure control, cholesterol management, and smoking cessation) measures, most primary care practices (PCPs) lack quality improvement (QI) support and resources to achieve meaningful targets. The Healthy Hearts for Oklahoma (H2O) Study proposes to build a QI infrastructure by (1) constructing a sustainable Oklahoma Primary Healthcare Improvement Collaborative (OPHIC) to support dissemination and implementation (D&I) of QI methods; (2) providing QI support in PCPs to better manage patients at risk for CVD events. Parallel to infrastructure building, H2O aims to conduct a comprehensive evaluation of the QI support D&I in primary care and assess the relationship between QI support uptake and changes in ABCS measures.

**Methods:**

H2O has partnered with public health agencies and communities to build OPHIC and facilitate QI. H2O has 263 small primary care practices across Oklahoma that receive the bundled QI intervention to improve ABCS performance. A stepped-wedge designed is used to evaluate D&I of QI support. Changes in ABCS measures will be estimated as a function of various components of the QI support and capacity and readiness of PCPs to change. Notes from academic detailing and practice facilitation sessions will be analyzed to help interpret findings on ABCS performance.

**Discussion:**

H2O program is designed to improve cardiovascular health and outcomes for more than 1.25 million Oklahomans. The infrastructure established as a result of this funding will help reach medically underserved Oklahomans, particularly among rural and tribal populations. Lessons learned from this project will guide future strategies for D&I of evidence-based practices in PCPs. Trained practice facilitators will continue to serve as critical resource to assists small, rural PCPs in adapting to the ever-changing health environment and continue to deliver quality care to their communities.

**Electronic supplementary material:**

The online version of this article (10.1186/s12913-018-3189-4) contains supplementary material, which is available to authorized users.

## Background

Cardiovascular disease (CVD) is the leading cause of death in the US and accounts for 17% of national health expenditures. Each year, more than two million adults in the US experience a heart attack or stroke, with more than 800,000 dying from CVD [1]. By 2030, 40.5% of the US population is projected to have some form of CVD and between 2010 and 2030, total direct medical costs of CVD are projected to triple from $273 billion to $818 billion, with indirect costs, due to lost productivity, increasing from $172 billion to $276 billion [2].

Specific to the state of Oklahoma, which has ranked near the bottom across a host of health indicators, the problem is even more alarming. CVD is the leading cause of death among Oklahomans [3, 4]. Based on the 2016 United Health Foundation rankings, Oklahoma has the third highest CVD mortality rate in the US, with 325.9 CVD deaths per 100,000 population, compared to Minnesota, which has the lowest CVD death rate, with 188.2 CVD deaths per 100,000 population [3]. In 2014, there were 5256 deaths among males and 4613 deaths among females attributed to heart disease in Oklahoma [4]. The percentage of preventable CVD deaths is particularly high among minority subgroups, with 65–70% of death among Black, American Indian, Asian/Pacific Islander, and Hispanic males classified as preventable compared to roughly 45% of deaths among non-Hispanic Caucasian males [3]. Percentages of preventable CVD deaths are lower among females, but the same trends are evident with 45–50% of deaths among Black, American Indian, Asian/Pacific Islander, and Hispanic females classified as preventable compared to approximately 25% among non-Hispanic Caucasian females [3].

In response to the CVD burden and alarming projections of CVD-related morbidity, mortality, and costs, the Department of Health and Human Services, along with other government and private agencies, launched the *Million Hearts Initiative* in 2011. The goal of the *Million Hearts Initiative* is to prevent one million heart attacks and strokes by 2017 through a focus on community- and clinic-based strategies to manage “ABCS” – aspirin for high-risk patients, blood pressure control, cholesterol management, and smoking cessation [1]. The initiative focuses on the implementation of proven, effective, and inexpensive interventions with two primary targets: (1) Improve clinical management of low-dose aspirin use, blood-pressure control, cholesterol management, and smoking cessation; and (2) Expand community initiatives to reduce smoking, improve nutrition, and reduce blood pressure. Ford et al. investigated the impact of surgical and medical treatments, relative to the reduction in coronary risk factors, on coronary deaths between 1980 and 2000. They found that while 47% of the reduction in the age-adjusted death rate for CVD could be attributed to medical and surgical treatments of cardiovascular events, 44% could be attributed to changes in risk factors, including reductions in total cholesterol, systolic blood pressure control, reduced smoking prevalence, and increase in physical activity [5]. Recommended strategies to improve adherence to ABCS guidelines include “team-based care, patient-centered medical homes, use of health information technology (HIT), and interventions to promote adherence.” These interventions should be “supported, evaluated, and disseminated rapidly to increase use of effective ABCS care practices [1].”

While ABCS guidelines are well defined, adherence in the clinical setting is less than optimal. National data from the *Million Hearts Initiative* suggests that 54% of individuals at increased risk of CVD events are taking aspirin, 53% of those with hypertension have adequately controlled blood pressure, 32% of individuals with high cholesterol are effectively managed, and 22% of people trying to quit smoking get counseling or treatment [6]. Furthermore, a large number of US adults are unaware of their high blood pressure and high cholesterol. Based on data from the 2003–2010 National Health and Nutrition Examination Survey, investigators estimated that among the 66.9 million US adults aged 18 years and older with hypertension, 14.1 million (39%) are not aware of their hypertension, 5.7 million (16%) are aware of their hypertension but are not receiving pharmacologic treatment, and 16.0 million (45%) are aware of their hypertension and are being treated with medication [7]. Similarly, a large percentage of adults are unaware of their high cholesterol status, at approximately 40%, and this rate is nearly 50% among Hispanic adults [8, 9]. While risk factor reduction, focused on ABCS measures, is recognized as a valuable approach to reducing CVD deaths, adherence to ABCS screening and treatment guidelines in primary care is deficient, with high percentages of patients unaware of their elevated risk status and clinicians juggling competing demands and prioritizing more acute illness over preventive screenings. New strategies to engage patients in and provide resources to practices for ABCS screening and management are urgently needed to address these challenges.

The Healthy Hearts for Oklahoma (H2O) Study, one of the seven collaboratives funded by the Agency for Healthcare Research and Quality (AHRQ) EvidenceNOW Initiative, proposes to build a quality improvement (QI) infrastructure in the state by (1) constructing a sustainable Primary Healthcare Improvement Center (OPHIC) that serves as a resource to support dissemination and implementation (D&I) of QI methods in Oklahoma; (2) facilitating the implementation of a bundled QI intervention in primary care practices to improve the management of patients at risk for CVD events. Parallel to infrastructure building, H2O aims to conduct a comprehensive evaluation of the bundled QI intervention implementation in primary care and hypothesizes that the QI intervention is associated with improvement in ABCS measures.

## Methods

### QI infrastructure

#### Resource center

H2O is developing a statewide D&I resource center, OPHIC, located within the state’s only comprehensive academic health center, with the capability to track patient-centered outcomes research (PCOR) results, assess needs of practices and communities, and provide corresponding D&I support to community clinicians and practices.

Specific to H2O, OPHIC is tasked with providing QI and medical informatics support to primary care practices. The OPHIC QI staff includes Practice Facilitator Coordinators (PFCs), Practice Facilitators (PFs), Academic Detailers (ADs), and HIT Regional Extension Center-Practice Advisors (REC-PAs). OPHIC recruits, trains, and certifies these personnel in QI methods. As the success of increasing QI capacity in small practices relies on automated data collection, performance reporting, and tracking, OPHIC, with its HIT consultants develops technical specifications for data collection and reporting, provides technical assistance to the PFs and REC-PAs to guide practices with clinical data capture in the electronic health records (EHR) that meets specifications to calculate clinical quality measures. OPHIC will also connect practices to knowledge and educational resources that will assist in planning QI activities using a listserv.

#### Analysis

Descriptive statistics will be used to summarize the number of personnel and time required to recruit, train, and deploy a sufficient number of ADs and PFs to support H2O throughout the state. Retention of personnel and geographic coverage areas of ADs and PFs, over the course of the study, will be summarized.

### Implementation of multi-component QI support strategy

#### QI implementation support strategy

**Fig. 1:**
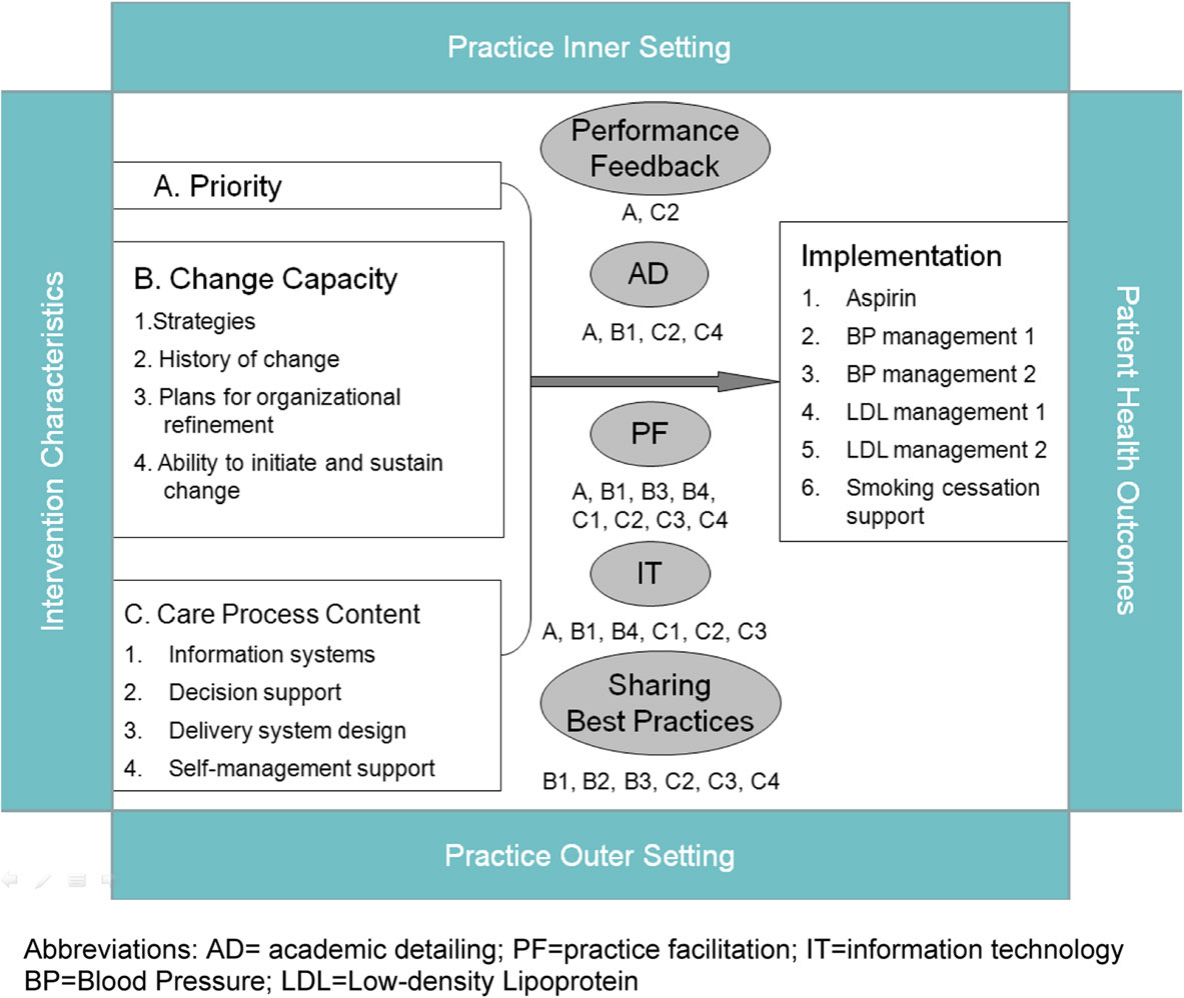
Conceptual model for implementation strategy

H2O delivers to each participating practice the following QI intervention components: (1) academic detailing provided by a primary care physician, (2) baseline and monthly performance feedback, (3) practice facilitation provided by a trained and certified PF, (4) Health Information Exchange (HIE) and HIT support, and (5) a Collaboration Website and listserv through which to share best practices. Figure 1 provides the conceptual model, anchored in Solberg’s Practice Change Model, that elucidates the likely effects of each component of the implementation support strategy on a practice’s QI priority, change capacity, and care process contents [10].

##### Academic detailing

Academic detailing has been shown effective for changing certain clinician behaviors including delivery of smoking cessation counseling [11] and appropriate use of antibiotics [12], though it was ineffective in increasing cervical cancer screening rates [13] and implementing depression management guidelines [14]. A Cochrane Collaboration review by O’Brien et al. concluded that “educational outreach visits, particularly when combined with social marketing, appear to be a promising approach to modifying professional behavior, especially prescribing [15].” H2O AD visits involve conversations with practice clinicians and staff about: 1) evidence; 2) current practice; and 3) characteristics of high performing practices. Academic detailing begins with a kick-off meeting to elicit a preliminary QI plan for the practice. The AD uses evidence-based summaries and decision support tools in their work with the practice. The ADs will make at least two visits to each practice during the intervention period and stay in contact with the practice throughout the project.

##### Performance feedback

Performance feedback has been demonstrated as one of the most effective mechanisms to motivate clinicians and practices to change [16–19]. Performance feedback for this study is provided in two ways. First, reports are generated from the HIE and/or EHR based upon patients’ meeting ABCS performance benchmarks. The practices receive baseline performance reports and then monthly reports for 1 year post implementation of QI strategy. Second, we will disseminate “best practices” from high performing practices and share “lessons learned [20–24].”

##### Practice facilitation

Practice facilitation has proven useful for helping primary care practices with implementation of new processes of care [25–27]. PFs embedded in the practice act as “change agents” and facilitate individualized solutions through rapid plan-do-study-act QI cycles. The presence of a PF also serves as a reminder of the practice’s commitment to make changes and increases their capacity to do so [28]. Assumptions inherent in the use of PFs in primary care are that many primary care practices are inadequately resourced, lack the experience and skills to sustain a major QI initiative, and are so different from one another that implementation must be customized. The relationships established by the PF with members of the practice appear to be critical to their effectiveness [29]. While facilitation is more expensive than most other QI approaches, reductions in inappropriate testing may more than offset these costs. For example, Hogg’s work showed a 1.4 return on investment on implementing preventive services [30].

##### HIE and HIT support

Advanced information systems will be required to provide ABCS performance reports [31]. H2O personnel help practices make more effective use of their EHRs and participate in HIEs. The REC-PAs visit each practice on a as needed-basis during the intervention period to help practices maximize electronic documentation and reporting of ABCS data, and train practices to use HIE to generate performance reports.

##### Collaboration website and listserv

The website will include dashboard pages for each participating practice and county, which will be used primarily by practices and PFs. There will also be a set of project pages displaying de-identified, comparative run charts and other project data as well as resources and resource links. The listserv will be updated on a weekly basis with questions, tips, and resource links.

#### Practice recruitment and enrollment

**Fig. 2:**
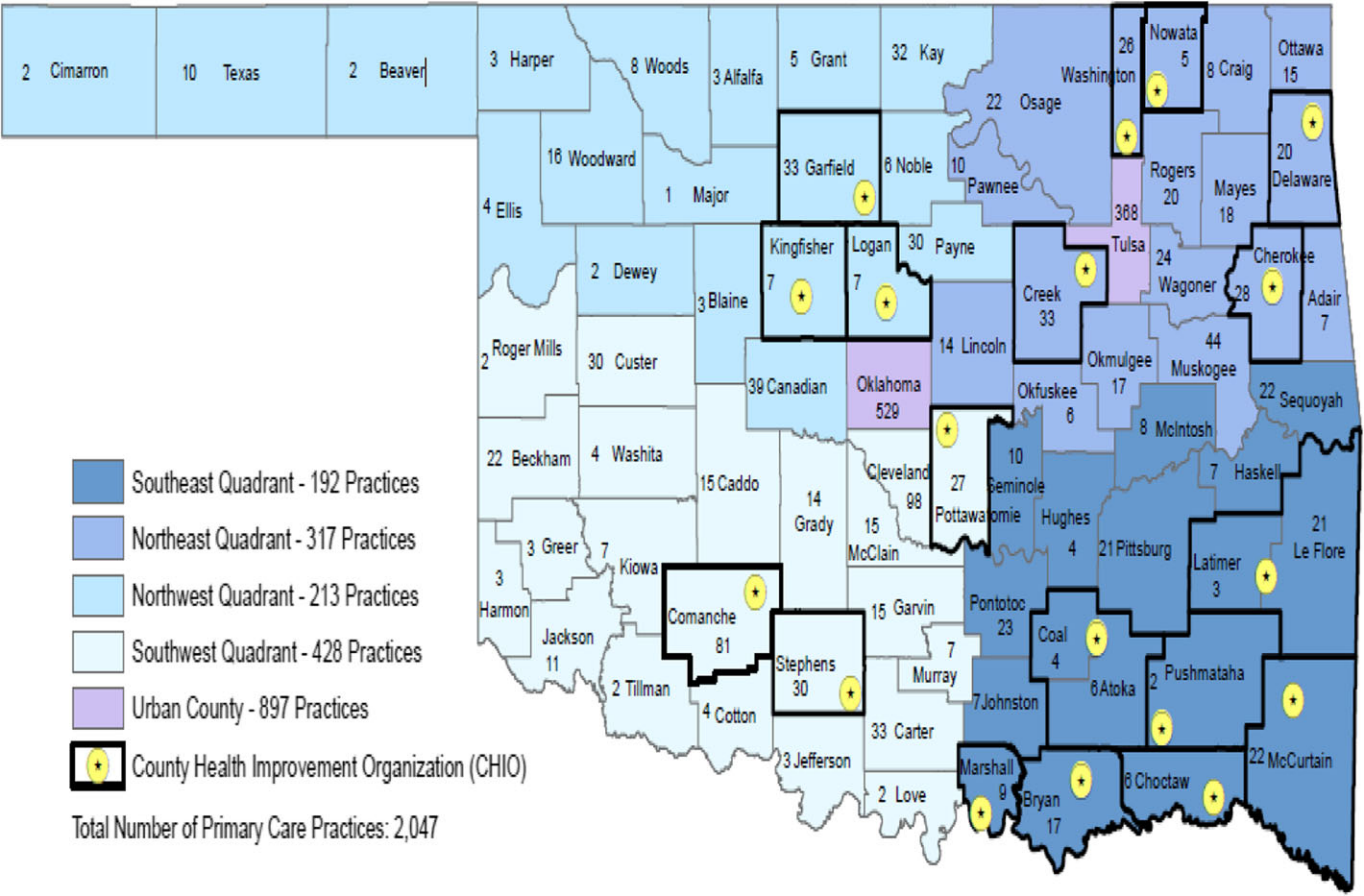
Primary care practices servicing adults in Oklahoma Counties

Figure 2 presents the location of primary care practices in Oklahoma by county. In total, it is estimated that Oklahoma has 2047 primary care practices that care for adults, but fewer than 25 have more than 10 primary care clinicians. There are 46 Medicare certified Rural Health Clinics [32], 17 community health centers providing services with 58 sites [33], 52 Indian Health Services or American Indian tribal clinics, two Department of Defense clinics, 13 Department of Veterans Affairs clinics, and 75 free clinics. Only practices with an EHR and a willingness to connect to the HIE were eligible to participate in H2O.

To recruit these practices, H2O collaborated with professional associations, health systems, payers, the practice-based research network, and the Oklahoma City Area Inter-Tribal Health Board. Incentives for participation included: (1) updates on the new blood pressure and lipid guidelines and ABCS decision aids; (2) in-practice QI support to enhance capacity, (3) assistance with Physician Quality Reporting System requirements for Medicare incentives, (4) credits for MOC Part IV and for Continuing Medical Education, (5) assistance achieving Meaningful Use of EHR certification for enhanced payment, (6) assistance qualifying for the Medicare Transition of Care and Care Coordination payments, and (7) reimbursement for expenses relating to the evaluation component. Moreover, for practices in counties with a County Health Improvement Organization (CHIO), H2O worked with the CHIOs to provide an incentive of $1000 for each participating practice to use for county-wide cardiovascular risk reduction campaigns. A PF contacted interested practices to arrange a kick-off visit to complete the enrollment process.

#### Analysis

Descriptive statistics will be used to summarize the deployment of the implementation support strategy among the enrolled practices over the course of the study. The extent to which the implementation support strategy is implemented at each practice will be quantified by the PFs based on categorical (qualitative) assessment variables.

### Evaluation

A comprehensive, systematic evaluation of the implementation strategy among participating primary care practices will be conducted to assess uptake of the implementation strategy as well as practice performance and outcomes.

#### Logic model

**Fig. 3:**
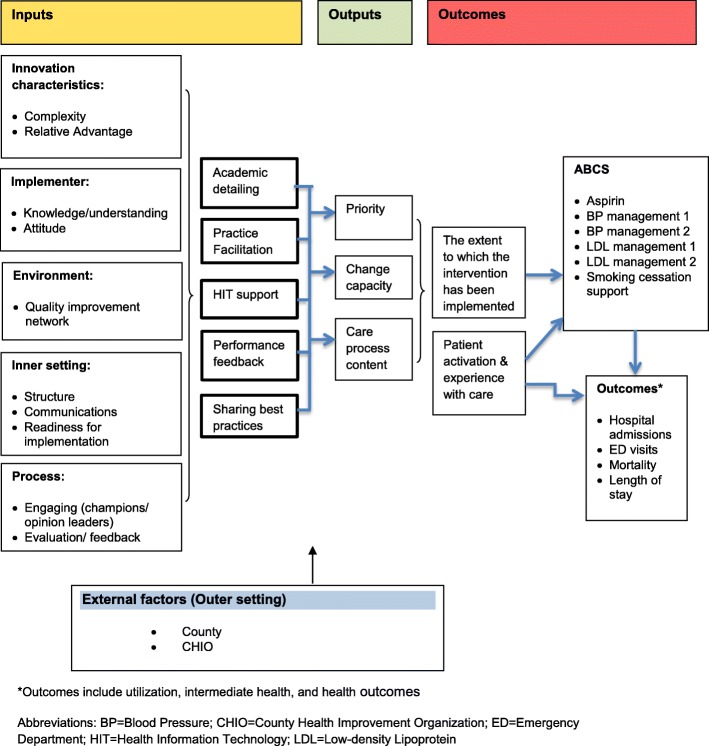
Logic model

Figure 3 presents the overall logic model guiding the evaluation. The model includes the following components: inputs, outputs, external factors, and outcomes.

Inputs include components of the implementation support strategy and organizational contextual factors that may facilitate or impede their uptake. Categories of inputs are adapted from the Consolidated Framework for Implementation Research (CFIR). CFIR [34] has grouped these factors into a number of domains, providing a “menu” for managers and operations leaders from which to select those that fit the particular setting and situation to explain QI initiatives, guide diagnostic assessments of implementation, and evaluate implementation progress and outcomes. Using CFIR, we aim to identify contextual factors that affect other inputs, outputs, and outcomes in the following domains: (1) intervention characteristics, (2) characteristics of the individual implementers, (3) inner setting, (4) environment; and (5) process of implementation (Table 1).

**Table 1:** Definitions for Consolidated Framework for Implementation Research (CFIR) Domains

CFIR Domain	Definition
Innovation characteristics	Innovation characteristics include the innovation itself, evidence strength and quality, relative advantage, complexity, design quality and packaging, etc. The innovation in this context is the implementation support strategy. For the purpose of this project, we will measure two characteristics of the implementation support strategy: complexity and relative advantage.
Characteristics of the individual implementer	Characteristics of clinicians and staff in a given practice who implement the support strategy include knowledge and understanding of the strategy, mindfulness, and personal attributes such as attitude, motivation, values, competency capacity, and learning style.
Inner setting	Organizational structure and mechanisms describe practices’ teamwork and communications, organizational culture, climate and readiness for implementation. Organizational climate illustrates practices’ tension for change, relative priority, incentives and rewards, goals and feedback, and learning.
Environment	The environment takes into account the location where the practice is situated, and the practice’s relationships with other organizations such as membership in a quality improvement network, health system, or professional society.
Process of implementation	The implementation process involves 4 stages: planning, engaging, executing, and reflecting and evaluating. Practices will work with ADs and PFs to select scheme, methods, and tasks for implementing the ABCS during the planning stage. The planning is followed by engaging the opinion leaders, internal implementation leaders, champions, and external change agents. The implementation plan is executed and evaluated with quantitative and qualitative feedback about the progress and quality of implementation.

*Abbreviations*: *ABCS* aspirin, blood pressure, cholesterol and smoking measures, *AD* academic detailer, *PF* practice facilitator

Outputs from the implementation support strategy, facilitated by organizational contextual factors, are the three requirements for improvement identified in the conceptual framework: (1) priority for change; (2) change process capability; and (3) care process content [10]. First, as practices face competing priorities, a practice must identify a specific QI initiative that will most benefit the practice’s mission and be supported by sufficient resources, staff commitment, and buy-in. Second, a practice must have the capacity and capability to change. This might include a culture that supports innovation, regular QI team meetings, ability to generate performance reports, and taking pride in seeing outcomes improve [10, 35, 36]. Third, “care process content” refers to processes such as delivery system design, decision support, and information systems, etc. as well as any specific resource required to improve a particular process. Addressing each of the output components would result in significant, sustainable improvements in quality of care.

Inputs and outputs are influenced by external factors or outer setting, such as characteristics of the county in which the practice is located and community resources (e.g., the availability of a CHIO). Inputs, outputs, and external factors, all affect outcomes. This evaluation aims to examine two sets of outcomes: (1) the extent to which the interventions have been implemented, as measured by ABCS practice performance; and (2) patient-oriented health outcomes (e.g., including utilization of EDs and hospitals, cardiovascular events, and deaths).

#### Sampling methods

**Fig. 4:**
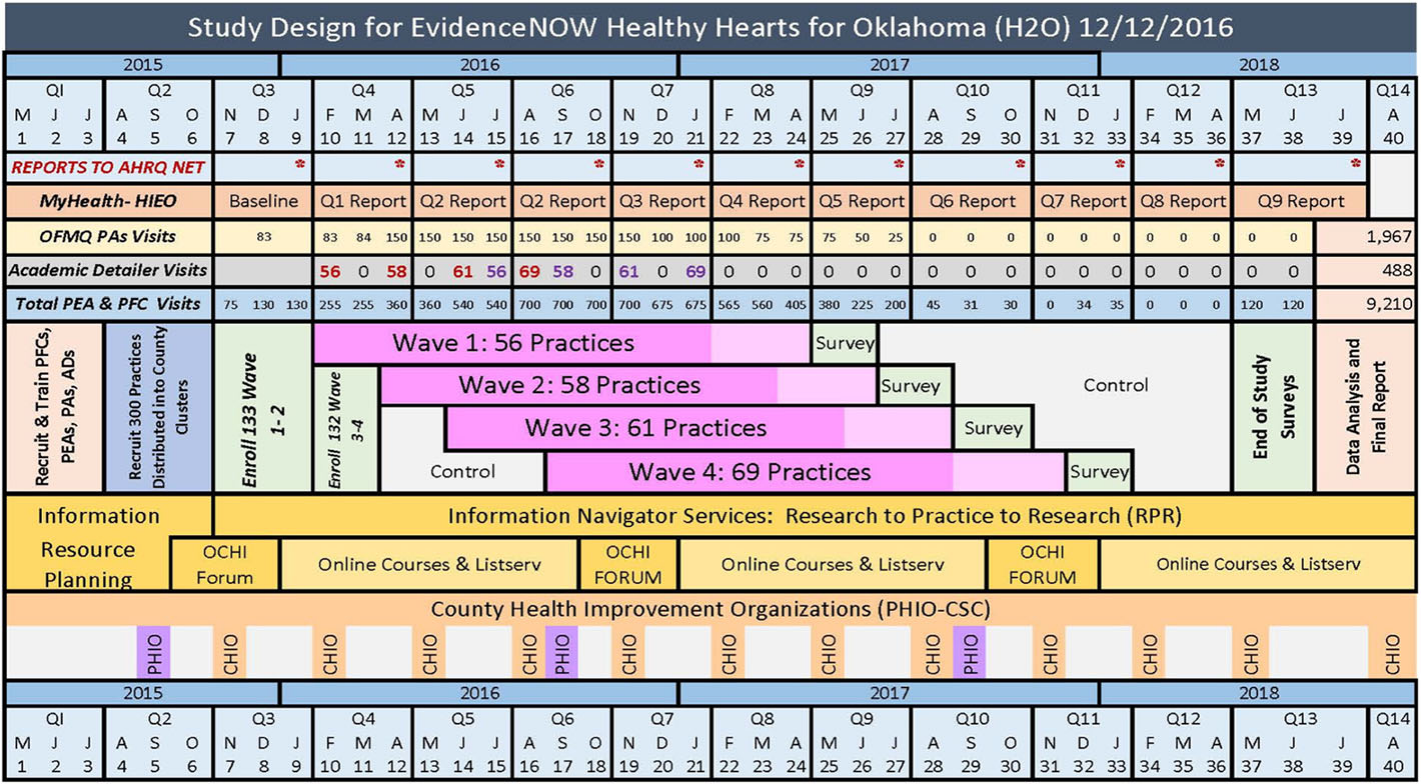
Stepped wedge cluster randomized study design

The original design included a targeted enrollment of 300 practices, but was revised to reflect a target of 250 practices early in the implementation phase to address feasibility concerns. According to the design, the practices are nested within counties, which are nested within 5 geographic sectors (4 quadrants of the state plus 2 metropolitan areas of Oklahoma and Tulsa counties). A total of 50 practices were sampled per quadrant and 25 practices were sampled per metro area (Fig. 2). The quadrant area boundaries were based on the Area Health Education Centers (AHEC) boundaries [37]. When developing the sampling scheme, a convenience sample of practices was drawn from within each county with the exception of counties that share a CHIO. Counties that share a CHIO were considered a single sampling unit; therefore, practices were sampled from each of the 75 counties or paired county units. At the completion of recruitment in November 2015, a total of 263 practices consented and were recruited (Fig. 4).

#### Evaluation design

A stepped-wedge cluster randomized trial design was used to evaluate the program. ABCS outcomes would be evaluated every 3 months beginning in month 7 of the project following an initial 6-month period for recruitment of the practices and practice units within each county, validation of the HIE data, and development of computing code for data abstraction from the HIE system. ABCS performance will be evaluated at each of the 263 practices during each 3-month period where the time of initiation of implementation support is randomly assigned. Each of 20 PFs were assigned to geographic sub-regions, nested within the five geographic sectors, in the state. The sub-regions reflected collections of counties that were feasible to access by a given PF. Within each sub-region, the consenting practices were randomly assigned to begin the intervention program in Wave 1, 2, 3, or 4 (Fig. 4). The targeted randomization was a total of 13 randomized practices per PF with three to four practices initiating the intervention program per Wave per PF. Random assignments were made in a manner to ensure a balance of Wave assignments for each PF and sub-region. In addition, during the 12-month implementation support period, two of the four programs were introduced in each 6-month period, where blood pressure management and smoking cessation support were introduced together and aspirin use and cholesterol management were introduced together. Within each of the four Waves, half of the counties introduced blood pressure management and smoking cessation support first and the other half introduced aspirin use and cholesterol management first, where the order assignment was randomly determined to ensure a balance by PF sub-region. The randomization sequences were generated using random number generation in Excel.

#### Surveys

Guided by the logic model, three survey instruments have been developed to identify inputs, implementer characteristics, inner setting, and process that may be associated with each practice’s readiness to change. The first instrument, Practice Characteristics Survey, collects practice demographic information as well as responses to validated questions from the Change Process Capacility Questionnaire (CPCQ) and National Ambulatory Medical Care Survey (NAMCS). The 32-item CPCQ assesses outputs by measuring three componenets of change capacity [38, 39]. The 25-item NAMCS assesses the degree of EHR adoption and functionalities at each practice (Additional file 1). To assess external factors and the environment, we document the county in which the practice is located and if the county has a CHIO to facilitate QI and if the practice is a member of a current or previous QI network. Additionally we use the “5Ps Microsystem Dashboard” developed by Dartmouth Institute for Health Policy and Clinical Practice to describe the practice. This survey is completed by practice leadership/management.

The second instrument, the Practice Members Survey, assesses perceptions of the change process and context among clinicians and staff using the 23-item Adapative Reserve questionnaire. Adaptive Reserve includes items that demonstrate a practice's ability to make and sustain change, such as practice relationship infrastructure, alignment of management functions in which clinical care, practice operations, and financial functions share and reflect a consistent vision, leadership, teamwork, work environment, and culture of learning [40]. This survey is completed by multiple members of the same practice who occupy the clinical/administrative hierarchy (Additional file 2).

The third instrument, Electronic Practice Record, assesses utility of implementation support strategy. PFs and ADs contribute their notes to structured and semi-structured items during facilitation and academic detailing sessions for each of ABCS metrics.

#### Data sources

The H2O master dataset will include both primary and secondary data sources. The aforementioned surveys will be compiled to include variables describing organizational contexts and outputs. These data are collected by PFs during visits to practices at three time points: (1) baseline, (2) at the end of the implementation of support strategy, and (3) 6 months post-implementation. Secondary data on performance (e.g., ABCS) and patient-oriented outcomes (e.g., hospitalization, length of stay) is extracted from the EHRs and the HIE. Practice performance, i.e., the number of patients who achieved and provider performance on these measures, will be calculated for each practice (Table 2).

**Table 2:** Outcome measures from HIE and/or EHR data

Measure (Source)	Numerator	Denominator*	Exclusion	Data Source
Aspirin (PQRS 204/NQF 0068)	Patients in denominator with documented use of aspirin or other antithrombotic	Patients 18+ years of age with Ischemic Vascular Disease diagnosis, or hospital discharge for acute myocardial infarction, coronary artery bypass graft, or percutaneous coronary interventions	On another anticoagulant, GI bleeding history, aspirin allergy	Health Information Exchange (HIE)
Blood Pressure Management 1 (PQRS 236/NQF 0018)	Patients in denominator whose blood pressure was adequately controlled (< 140/90)	Patients aged 18 through 85 years with a diagnosis of hypertension	End stage renal disease, dialysis, renal transplant, or diagnosis of pregnancy	HIE
Blood Pressure Management 2	Patients in denominator whose blood pressure was adequately controlled (age 18–59 and/or people with diabetes or chronic kidney disease < 140/90; age 60–85 < 150/90)	Patients aged 18 through 85 with a diagnosis of hypertension	Lacking a DM or CKD diagnosis	HIE
Cholesterol Management 1 (PQRS 316)	Patients in the denominator whose risk-stratified fasting LDL is at or below the recommended LDL goal	Patients aged 20 through 79 years of age who had a fasting LDL performed		HIE and chart audits
Cholesterol Management 2	Patients in the denominator who were prescribed the recommended dose of statin based on risk status	Patients aged 20 through 79 years of age who had a fasting LDL performed		HIE and chart audits
Smoking Cessation Support (PQRS 226/NQF 0028)	Patients in denominator who were screened about tobacco use one or more times within 24 months AND who received tobacco cessation counseling intervention if identified as a tobacco user	Patients age 18+ years	Documentation of medical reason(s) for not screening for tobacco use	HIE

^*^Note: Population denominators will be based on active patient—defined as patients who have been seen in the practice within the previous 18 months

*Abbreviations*: *CABG* coronary artery bypass grafting, *CKD* chronic kidney disease, *COPD* chronic obstructive pulmonary disease, *CVA* cerebral vascular accident, *DM* diabetes mellitus, *ED* emergency department, *GI* gastric intestinal, *LDL* low-density lipoprotein, *LOS* length of stay, *MI* myocardial infarction, *PQRS* physician quality reporting system, *NQF* National Quality Forum

#### Power analysis and effect sizes

A series of simulation studies were performed to investigate power of the planned evaluation study [41]. A logistic regression model was used to randomly generate data corresponding to the assumed percentage of patients satisfying each ABCS criterion prior to and after the implementation support period as specified in Table 3. The model was specified according to the mean model introduced by Hussey and Hughes [42] and included a random effect corresponding to practice (the unit of randomization), a fixed implementation support effect where the implementation support covariate was modeled using a fractional covariate of (0.25, 0.5, 0.75, and 1.0) for the 3, 6, 9 and 12-month post-initiation practice performance measures to account for an implementation support strategy that is not fully effective until the 12-month time point following initiation. Note that for half of the practices, the fractional covariate is delayed by an additional 6 months relative to the Series implementation scheme given that one pair of targets (blood pressure/smoking or aspirin/cholesterol) is delayed by 6 months in each practice. The model also included a random error term. The correlation among measures made on patients from the same practice was assumed to be 0.10 or less [43]. After the data were generated, Generalized Estimating Equations (GEE) methodology was used to fit a logistic regression model to test the significance of the implementation support strategy effect. The percentage of simulated data sets that resulted in a significant intervention effect was recorded as the power of the specified study design. The power analysis assumes a 2-sided 0.05 alpha level. For each ABCS endpoint, 500 simulated data sets were generated. Estimated power is summarized for each target in Table 3. The power of the implemented analyses will be higher than that from the simulation studies, given that all active patients in the targeted clinical population for a particular ABCS criterion during a 3-month period will be analyzed per practice unit.

**Table 3:** Summary of the assumed intervention effect sizes and power

Endpoint	Patient Cohort Characteristics (defining cohort eligible for intervention)	Baseline^a^ Percentage	Post-intervention Percentage	Power
A: Aspirin (aspirin or other antithrombotic prescribed)	Aged 18 years and older with ischemic vascular disease without a contraindication to aspirin	60%	70%	> 95%
B1: Blood Pressure Management 1 (blood pressure adequately controlled)	Aged 18–85 who had a diagnosis of hypertension	65%	70%	> 95%
B2: Blood Pressure Management 2 (blood pressure adequately controlled as per co-morbidity-adjusted targets)	Aged 18–85 who had a diagnosis of hypertension	65%	70%	> 95%
C1: Cholesterol Management 1:** (fasting LDL test AND ≤ LDL goal)	Aged 20–79	15%	25%	> 95%
C2: Cholesterol Management 2:** (fasting LDL test AND prescribed statin if indicated)	Aged 20–79	15%	25%	> 95%
S: Smoking cessation support (screening about tobacco AND received cessation counseling if tobacco user)	Aged 18 years and older	60%	70%	> 95%

^a^Estimates for the baseline percentages for aspirin use, blood pressure management, and smoking cessation support are based on Oklahoma Foundation for Medical Quality (OFMQ) primary care practice initiatives in the state of Oklahoma

^**^The C1 and C2 measures were calculated as the product of the probability of having a fasting LDL test (estimated to be 0.3) multiplied by the probability of having an LDL measure below the target (estimated to be 0.50) or being prescribed a statin if indicated (estimated to be 0.5) conditional on having a fasting LDL test

*Abbreviation*: *LDL* low-density lipoprotein

Practices drop out of QI projects when there is a significant disruption in the practice’s internal (e.g., significant clinician or staff turnover, new EHR) or external (e.g., new ownership) environment. On average, we have experienced a drop-out rate of about 5% during a 6-month initiative from previous experience. We therefore estimated a 10% drop-out rate in this 1-year project. Among the 263 recruited practices, we expect to retain at least 234 practices for the entire duration of the 3-year evaluation program.

#### Analytic plan

The evaluation plan has 2 analytic goals: (1) determine the impact of the implementation support strategy on the practice’s capacity to change, performance indicators and patient outcomes; and (2) assess the role of internal and external contextual factors and intervention characteristics on the uptake of the implementation support strategy and on practice performance and outcomes.

1. Impact of the implementation support strategy on the practice’s capacity to change, performance indicators and patient outcomes

The effect of the implementation support strategy on the mean capacity to change score (priority for improvement, change capacity, and care process content), or the log odds of dichotomous indicators of change, will be estimated using GEE to fit linear and logistic regression models, respectively, based on a within-cluster and between-cluster analysis [42]. As discussed by Hussey and Hughes [42], GEE has several attractive properties that are relevant to the analysis of data arising from stepped wedge designs, including accommodating generalized linear models that are appropriate for continuous, count and dichotomous outcome measures, as well as robustness to variance and covariance misspecification. The small sample limitations of GEE-based estimation will not be of concern in this study given that 263 practices will serve as the unit of randomization and 11 time points will be utilized in the design. The outcome variable will be a continuous score measure or a Yes/No indicator variable measures at a practice unit level. The exposure of interest will be a dummy variable indicating the implementation support strategy exposure, which will be coded according to the randomized assignment (intent-to-treat analysis). Practice unit-level data will be analyzed where the nested clustering of practice units nested within practices, nested within sub-regions will be accounted for using a structured working correlation structure [44]. Cluster sizes, by county, will vary due to the targeted sampling of metro counties (25 practices per county) and more rural counties (one to five practices per county) and therefore, a jackknife estimate of the variance will be used to maintain the size of the test [42]. The time since the initiation of the implementation support strategy will also be considered as an independent factor in the regression model, along with a time by implementation support strategy interaction, to investigate the effect of the implementation support strategy relative to the time since initiation (i.e., delayed treatment effect). If a significant time by implementation support strategy effect is found, the analyses will be stratified by time period to estimate the time-specific implementation support strategy effect. Time-varying secular trends will be considered in the model, including the implementation of other health intervention programs in the studied sub-regions. Geographic region will also be considered as a modifying factor where region is fit as a series of four indicator variables (indicator for each quadrant, with the combined Tulsa and Oklahoma City metro areas serving as the reference group). If appropriate based on estimates, a dichotomous region variable indicating metro versus non-metro locations will be considered. A Type III Wald statistic will be used to determine the overall significance of the interaction between implementation support strategy and the five degrees of freedom categorical class variable for region. A 2-sided 0.05 alpha level will be used to define statistical significance. An intention-to-treat paradigm will be followed where data from all practice units are analyzed according to the randomized assignment regardless of adherence or implementation quality.

Logistic regression models will be fit to estimate the effect of the implementation support strategy on the log odds of attaining an ABCS criterion for a given patient. The outcome variable will be a Yes/No indication of attaining an ABCS criterion measured at a patient level. Individual-level data will be analyzed where the nested clustering of patients within practice units, nested within practices and counties will be accounted for using a structured working correlation structure [44].

Poisson regression models, with a log link, will be used to estimate the effect of the implementation support strategy on the incidence rate of patient outcomes, (ED visits, preventable hospitalizations, heart attacks, strokes, GI bleeds, deaths, etc.), where practice unit data are summarized for each 3-month time period. The outcome will be the number of patient events reported among the active adult patients in a practice unit and the offset will be the total number of patient-months of time at risk during the 3-month time period for the practice unit. GEE will be used to account for the correlation among nested practice units within practices within counties and modifying factors will be investigated.

(2) Role of internal and external contextual factors and intervention characteristics on the uptake of the implementation support strategy and on practice performance and outcomes.

General regression modeling strategies described by Baron and Kenny [45] will be used to investigate modifying and mediating factors, as presented in the Logic Model, controlling for clustering. In brief, factors that modify the effect of the implementation support strategy will be indicated by significant interaction terms in the regression model. For example, organizational contextual factor scores will be investigated as modifying factors to determine if there is evidence that the effect of the implementation support strategy is greater among those practice units that have higher scores on the contextual factors. Mediating variables will be investigated by fitting a series of four regression models. First, the effect of the implementation support strategy on the health outcome measure will be estimated. Second, the association between the implementation support strategy (independent factor) and the potential mediating factor (outcome) will be estimated. Third, the association between the mediating factor (independent factor) and the health outcome variable will be estimated. Finally, the model will be refit to include the implementation support strategy and mediating term as independent factors with the health outcome measure modeled as the outcome variable. A change in the implementation support strategy coefficient, indicating a shift towards no effect on the outcome, with adjustment for the mediating factor will provide evidence of a mediating association.

## Discussion

### Impact

Oklahoma is among the poorest states in the nation, with a population of 3.85 million, 75.5% of which is white, 7.6% black, 9.0% American Indian, 1.9% Asian, and 9.3% Hispanic [46]. The state has 38 federally recognized American Indian tribes and the majority of its counties are rural. Most rural communities have a large proportion of vulnerable and underserved patients. Relevant to the CVD management goals of H2O, 23.3% of Oklahomans are current smokers, 35.5% have hypertension, 31.1% are physically inactive, and 32.2% are obese [47]. In a 2011 government report, the quality of health care in Oklahoma was rated as “weak,” particularly in chronic disease management [48]. The H2O study, leveraging the establishment of OPHIC, puts forth a coordinated and systematic effort to engage and provide QI resources to practices, in both rural and urban counties, that serve vulnerable populations. Moreover, one of the reasons accounting for poor QI is the lack of infrastructure and appropriate technology. H2O provides technical and financial assistance to help practices with EHR support and connection to a HIE.

Over the course of the study, PFs, ADs, and PFCs support practices assisting them in establishing QI processes, empaneling and risk stratifying their patients, and providing care coordination for those at the highest CVD risk. Such planned care has been demonstrated to reduce hospitalizations, ED visits, utilization, and use of specialists when more active primary care is provided. In addition, practices may have the opportunity to connect to the preventive services registry or implement enhanced referral systems, both available through the HIE. All of these strategies will likely remain with the practice after study completion.

Enrollment in H2O also positions practices to new payment opportunities with OPHIC’s potential capability to negotiate purchasing of advanced primary care services. Of the 263 practices, 195 will have 12 or more months at the end of the study period to implement these advanced primary care services and be prepared for a shared savings payment plan similar to that tested in the multi-payer Comprehensive Primary Care program, or to participate in an Accountable Care Organization.

### Limitations

Notwithstanding the benefits, the study notes a number of barriers that require mitigating strategies. First, there may be potential barriers for practices to participate. Primary care practices are often understaffed and face competing demands. They may experience difficulty meeting QI initiative deadlines and having their interest in sustaining QI wane over time. Automation of clinical measure data collection and reporting, on-site consultation by an AD for the clinicians, and the weekly presence or availability of a PF will make the uptake of QI efforts more feasible. Individualizing the implementation approach and routinely sharing lessons across practices may also enhance the practice’s readiness to change.

Second, a major barrier to QI has been the time and effort required to document practice performance and patient outcomes. Until recently, the only method of obtaining QI data was to perform individual chart abstractions of paper or electronic records. This is costly, time-consuming, and invasive, although if done by professionals, remains the gold standard. This barrier may be ameliorated with the use of EHR and connectivity to an HIE. The PFs and REC-PAs will provide technical assistance to practices without EHRs to implement the system, and to practices with EHRs to generate practice-level performance reports electronically. As EHRs are connected to a HIE, these reports should be more accurate because they can be based upon a numerator that includes services received at multiple sites of care and a denominator that includes the practice’s whole panel rather than a convenience sample.

Third, recruitment and deployment of PF and REC-PAs to ensure an effective geographic distribution may be barrier given the size of the state. To minimize travel time and maximize time in the practices, the hiring of PFs was strategic in terms of location selection from all quadrants of the state with the involvement of the AHECs [37], CHIOs, and practices.

## Conclusion

H2O creates an infrastructure that supports a market for practice- and community-oriented professional development/employment, and a well-tested feasible QI model that is well-aligned with the public health system in Oklahoma. The infrastructure and the QI model are likely to be sustainable, especially if the infrastructure may facilitate linkage between county health targets with healthcare financing structure for primary care through payment reform for value-based purchasing. Achieving the study aims will benefit a state by improving its poor health outcomes and providing additional resources.

## Additional files

Additional file 1: H2O Practice Characteristics Survey. (PDF 700 kb)

Additional file 2: H2O Practice Member Survey. (PDF 784 kb)

## Abbreviations

ABCS: Aspirin therapy, blood pressure control, cholesterol management, smoking cessation
AD: Academic detailers
AHEC: Area Health Education Centers
AHRQ: Agency for Healthcare Research and Quality
CFIR: Consolidated framework for implementation research
CVD: Cardiovascular disease
D&I: Dissemination and implementation
ED: Emergency department
EHR: Electronic health records
H2O: Healthy Hearts Oklahoma
HIE: Health information exchange
HIT: Health information technology
HITREC: Health Information Technology Regional Extension Center
NAMCS: National Ambulatory Medical Care Survey
NHANES: National Health and Nutrition Examination Survey
OPHIC: Oklahoma Primary Healthcare Improvement Center
ORCA: Organizational Readiness Change Assessment
PF: Practice facilitators
PFC: Practice facilitator coordinators
QI: Quality improvement
REC-PA: Regional Extension Center-Practice Advisors
