# Supplementary material for: Disseminating, implementing, and evaluating patient-centered outcomes to improve cardiovascular care using a stepped-wedge design: healthy hearts for Oklahoma
Source: BMC Health Serv Res. 2018 Jun 4;18:404. doi: 10.1186/s12913-018-3189-4 (PMC5987433; doi:10.1186/s12913-018-3189-4)
Supplement: Supplementary file 1 — H2O Practice Characteristics Survey. (PDF 700 kb) [file 12913_2018_3189_MOESM1_ESM.pdf]

OKLAHOMA Clinical and Translational Science Institute
Located at The University of Oklahoma Health Sciences Center

James W. Mold OPHIC Oklahoma Primary Healthcare Improvement Cooperative

H2O Practice Characteristics Survey

Please complete the following survey, which is designed to collect information about your practice for the EvidenceNOW Healthy Hearts for Oklahoma (H2O)study.

This survey has three sections:

1) General Information,

2) EHR, and

3) Improving Patient Care.

We suggest you designate an Office Manager or a Lead Clinician to complete this survey. We strongly encourage you to consult with others in your practice (e.g., Medical Director, Billing Manager) to obtain accurate information to complete this survey.

SECTION 1: General Information

Practice site ID: ______________________________

Practice name : ______________________________

Practice Zip code: ______________________________

Date: ____/____/_________ (mm/dd/yyyy)

Please indicate others in the practice that you consulted with to complete this survey?

(CHECK ALL THAT APPLY)

☐ Medical assistant
☐ Clinician (MD, DO, NP, PA)
☐ Front office staff
☐ Office manager
☐ Back office staff
☐ Nurse
☐ Other (please specify) ______________________________

SECTION 1: General Information

1. **Which of the following best describes your practice size?** (CHECK ONE)
   - ☐ Solo practice
   - ☐ 2-5 clinicians (MD, DO, NP, PA)
   - ☐ 6-10 clinicians
   - ☐ 11-15 clinicians
   - ☐ 16 or more clinicians

2. **Which of the following best describes your practice's ownership?**
   (CHECK ALL THAT APPLY)
   - ☐ Clinician-owned solo or group practice
   - ☐ Hospital/Health system owned
   - ☐ Health maintenance organization (e.g., Kaiser Permanente)
   - ☐ Federally Qualified Health Center or Look-Alike
   - ☐ Non-federal government clinic (e.g., state, county, city, public health clinic, etc.)
   - ☐ Academic health center / faculty practice
   - ☐ Federal (Military, Veterans Administration, Department of Defense)
   - ☐ Rural Health Clinic
   - ☐ Indian Health Service
   - ☐ Other (please specify): ____________________
   ____

3. **How many years has your practice been under the current ownership? Please round to the nearest year. If your practice has been under the current ownership for less than one year, please indicate that by checking that response option.**

   ______ years

   ☐ ***This practice has been under the current ownership less than one year***

4. **Which of the following describes your practice's specialty mix?**
   - ☐ Single-specialty
   - ☐ Multi-specialty

Please provide the number of practice members and their combined FTE for each of the following type of staff:

5. **Clinicians (MD, DO, NP, PA)**

   Number of physicians ______________________

   Combined physician FTE ___________________

6. **Clinical Staff (those providing direct patient care, e.g. RN, LPN, MA, CMA)**

   Number of Clinical Staff ___________________

   Combined Clinical Staff FTE ________________

7. **Office Staff (those supporting practice operations and NOT involved directly in patient care, e.g., receptionists, billing staff, data analyst, etc.)**

   Number of office staff _____________________

   Combined office staff FTE __________________

8. **Psychologist**

   Number of Psychologists ___________________

   Combined Psychologist FTE ________________

9. **Social Worker or Licensed Social Worker**

   Number of Social workers __________________

   Combined Social workers FTE ______________

10. **PharmD or Pharmacist**

    Number of PharmD or Pharmacist __________

    Combined PharmD or Pharmacist FTE______

11. **Other practice members**

    Number of other practice members _________

    Combined Other practice members FTE _________________________

SECTION 1: General Information

12. Have there been any of the following major changes in your practice in the last 12 months? (CHECK ALL THAT APPLY)

- ☐ Implemented a new or different Electronic Health Record (EHR)
- ☐ Moved to a new location
- ☐ Lost one or more clinicians
- ☐ Lost one or more office managers or head nurses
- ☐ Been purchased by or affiliated with a larger organization
- ☐ New billing system
- ☐ Other (please specify): _______________

13. Is your practice recognized or accredited as a patient-centered medical home (PCMH)?

- ☐ Yes
- ☐ No

14. Is your practice part of an accountable care organization (ACO)? (CHECK ALL THAT APPLY)

- ☐ Yes, Medicaid ACO ***(if checked, skip #15)***
- ☐ Yes, Medicare ACO (Pioneer, Shared Savings Plan, or Advance Payment) ***(if checked, skip #16)***
- ☐ Yes, Private/Commercial ACO
- ☐ Yes, Another type of ACO
- ☐ No, not part of an ACO
- ☐ Don't know

15. Do you plan on newly joining or newly contracting with a Medicaid ACO or ACO-type arrangement in the next 12 months?

- ☐ Yes
- ☐ No

16. If you are not part of a Medicare ACO, do you plan on newly joining or newly contracting with a Medicare ACO (Pioneer or Shared Savings Plan) in the next 12 months?

- ☐ Yes
- ☐ No

17. Do you plan on newly joining or newly contracting with a private/commercial ACO or ACO-type arrangement in the next 12 months?

- ☐ Yes
- ☐ No
- ☐ Already contracting with at least one Private/Commercial ACO but plan to join additional Private/Commercial ACO arrangements

18. Please estimate the total number of patient visits over a typical week at your practice.

Visits per week: ____________

19. Do the clinicians in your practice have their own panel of patients for whom they are responsible?

- ☐ Yes
- ☐ No **(SKIP to 21)**

20. Please estimate the average patient panel size for a full-time clinician in your practice.

Average panel size: ____________

21. On average, how many patients does a full-time clinician in your practice see on a typical day?

Average # patients seen: ___________

SECTION 1: General Information

Now, we would like you to answer a few questions about your practice's patients. For race, ethnicity and age, this information should be reported using the Electronic Health Record (EHR) /Practice Management System (PMS), if possible.

22. Please give the percentage of your patients in the following categories:
(SHOULD ADD TO 100%)

___________ White
___________ Black/African American
___________ American Indian or Alaska Native
___________ Asian
___________ Native Hawaiian or Other Pacific Islander
___________ Some Other Race/Mixed Race
___________ Percent Unknown
☐ Our practice does not collect this information from patients

The above values…

☐ Were obtained from patient self-report data and calculated using the Electronic Health Record (EHR) /Practice Management System (PMS)

☐ Estimates (please explain)_______________
____
____
____

☐ Other (please specify)___________________
____
____
____
____
____

23. Please give the percentage of your patients in the following categories:
(SHOULD ADD TO 100%)

___________ Hispanic or Latino
___________ Non-Hispanic or non-Latino
___________ Percent Unknown
☐ Our practice does not collect this information from patients

The above values…

☐ were obtained from patient self-report data and calculated using the Electronic Health Record (EHR) /Practice Management System (PMS)

☐ are estimates (please explain)___________
____

☐ other (please specify)___________________
____
____

24. Please give the percentage of your patients in the following age categories:
(SHOULD ADD TO 100%)

___________ 0-17
___________ 18-39
___________ 40-59
___________ 60-75
___________ 76 and over

The above values…

☐ were obtained from patient self-reported data and calculated using the Electronic Health Record (EHR) /Practice Management System (PMS)

☐ Estimates (please explain) ______________
____
____

☐ Other (please specify)___________________
____
____

25. **Please give the percentage of your patients who are male and female:** (SHOULD ADD TO 100%)

___________Male

___________Female

26. **Please give the approximate percentage of your patients in the following payer categories:** (should add to 100%)

_____________ Medicare only

_____________ Medicaid only

_____________ Dual Medicare and Medicaid

_____________ Private or commercial

_____________ No insurance

_____________ Other (please specify: _______

____

____

27. **Has your practice been designated as a medically underserved area (MUA) or medically underserved population (MUP) by the Health Resources and Service Administration (HRSA)?**

☐ Yes

☐ No

Now, we would like you to answer some questions about how your practice externally reports on clinical quality measures and how your practice payment may be adjusted according to performance on the measures.

28. **Are data on the clinical quality of care provided by your practice or its clinicians publicly reported by health plans or other external entities?**

☐ Yes

☐ No

☐ Don't know

29. **During meetings in your practice, how often - if ever - are these data or reports about clinical quality from health plans or other external entities discussed?**

☐ Never

☐ Infrequently

☐ Often

☐ Not applicable/Solo practice

☐ Don't know

30. **Does your practice work with the following organizations/networks to support capture of EHR/Electronic Medical Record (EMR) data used to report clinical quality measures?** (CHECK ALL THAT APPLY)

☐ Clinical data warehouse

☐ Regional extension center

☐ Health system practice network

☐ Health information exchange

☐ Primary care association

☐ Hospital network

☐ External consulting group

☐ Practice-based research network

Next, we would like to understand how your practice uses registries and clinical guidelines for cardiovascular disease prevention. Please consult with the lead clinician at your practice to answer these questions.

31. **Please indicate the categories of patients for which your practice uses a registry or receives reports that identify services due, gaps in care, or track progress:**

(CHECK ALL THAT APPLY)

☐ Ischemic vascular disease

☐ Hypertension

☐ High cholesterol

☐ Diabetes

☐ Prevention services

☐ High risk (high utilization) patients

☐ We do not use registries or receive such reports

32. **Please identify how your practice uses clinical guidelines for cardiovascular disease prevention (for example, use of aspirin or antithrombotics for those with ischemic vascular disease or smoking cessation counseling):**

(CHECK ALL THAT APPLY)

☐ Practice does not follow specific guidelines

☐ Guidelines are posted or have been distributed

☐ Clinicians have agreed to use specific guidelines

☐ Practice uses standing orders

☐ Practice uses EHR provider guideline-based prompts and reminders

33. **Please identify how your practice uses clinical guidelines <u>for management of patients at risk for cardiovascular disease</u> (e.g., statin use among those at risk):**

(CHECK ALL THAT APPLY)

☐ Practice does not follow specific guidelines

☐ Guidelines are posted or have been distributed

☐ Clinicians have agreed to use specific guidelines

☐ Practice uses standing orders

☐ Practice uses EHR provider guideline-based prompts and reminders

34. **Over the past 12 months, did your practice or individual clinicians in your practice receive additional revenue or payments from a health plan, public payer, or health system based on measurement of performance on the following measures?**

a. **Measures of patient satisfaction**

☐ Yes

☐ No

☐ Don't know

b. **Measures of clinical quality**

☐ Yes

☐ No

☐ Don't know

c. **Measurement of your performance of adoption or use of information technology**

☐ Yes

☐ No

☐ Don't know

35. Over the past 12 months, did your practice receive better contracts (for example, better payment, preferred status) with health plans for its performance on measurements of patient satisfaction and/or clinical quality?

☐ Yes

☐ No

☐ Don't know

36. Over the past 12 months, did your practice or the individual clinicians in your practice receive additional income from health plans based on efficient utilization of resources?

☐ Yes

☐ No **(SKIP to 38)**

☐ Don't know **(SKIP to 38)**

37. What percent of your practice's annual revenue did these additional payments for efficient utilization of resources constitute?

________%

☐ Don't know

38. Over the past 12 months has your practice received the following forms of bonus or incentive payments?

(CHECK ALL THAT APPLY)

☐ Geographic health care professional shortage area

☐Medicare primary care incentive payment

☐ Medicare care coordination payment

☐ Other (please specify):________________

____

39. At present or within the past 12 months, has your practice participated in any of the following payment or quality demonstration programs? (CHECK ALL THAT APPLY)

☐ State Innovation Models initiative (SIM)

☐ Comprehensive Primary Care Initiative (CPCI)

☐ Transforming Clinical Practice Initiative (TCPI) – Support and Alignment Network (SAM)

☐ Community Health Worker training program (CHW)

☐ BC/BS PCMH program

☐ ASTHO's Million Hearts State Learning Collaborative

☐ Million Hearts: Cardiovascular Disease Risk Reduction Model

☐ Other (please specify): ________________

____

____

Please continue to SECTION 2 on the next page.

SECTION 2: Electronic Health Record Information

These questions ask about your practice's use of an electronic health record (EHR) or electronic medical record (EMR) system. Please consult with the lead clinician at your practice to answer these questions.

40. Does your practice use an EHR system or EMR system? Do not include billing record systems.

☐ Yes, all electronic

☐ Yes, part paper and part electronic

☐ No **(SKIP to QUESTION 57)**

☐ Unknown **(SKIP to QUESTION 57)**

41. In which year did you install your current EHR/EMR system?

____________Year (yyyy)

42. What is the name of your current EHR/EMR system? (CHECK ONLY ONE BOX)

If "Other" is checked, please specify the name

☐ Allscripts

☐ AdvancedMD

☐ Amazing Charts

☐ Athenahealth

☐ Care360

☐ Cerner

☐ eClinicalWorks

☐ e-MDs

☐ EPIC

☐ GE/Centricity

☐ Greenway Medical

☐ McKesson/Practice Partner

☐ NextGen

☐ Practice Fusion

☐ Sage/Vitera

☐ SOAPware

☐ Other (please specify): ____________________

____

43. What version of your EHR/EMR system are you currently using? You should be able to find the software version on the log-in screen.

Version number: _______________

44. Do you share any patient health information (e.g., lab results, imaging reports, problem lists, medication lists) electronically (not fax) with any other providers, including hospitals, ambulatory providers, or labs?

☐ Yes

☐ No

45. Where does your data physically reside? (CHOOSE ONE OPTION)

☐ Data resides only on a server in your practice

☐ Data resides in a server in your practice and copy in the "cloud"

☐ Data resides in the "cloud" with vendor

☐ Data resides in health system data warehouse somewhere

☐ Do not know where data resides

46. Medicare and Medicaid offer incentives to practices that demonstrate "meaningful use of health IT."

Is your current EHR/EMR system certified to meet Meaningful Use as defined by Health and Human Services / Office of the National Coordinator for Health Information Technology (ONC)?

☐ Yes

☐ No

☐ Uncertain

47. Medicare and Medicaid offered incentives to practices that demonstrate "meaningful use of health IT." Did your practice apply for Stage 1 of these incentive payments?

☐ Yes

☐ No **(SKIP to 49)**

☐ Uncertain **(SKIP to 49)**

48. Are there plans to apply for Stage 2 incentive payments?

☐ Yes

☐ No

☐ Maybe

☐ Unknown

49. Is your practice able to incorporate clinical lab-test results into EHR/EMR as structured data (i.e., data recorded in discrete fields and not in text fields)?

☐ Yes

☐ No

50. Does your practice have someone who can configure or write quality reports from the EHR/EMR?

☐ Yes

☐ No **(SKIP to 52)**

51. Who is the person primarily responsible for configuring and writing quality reports from the EHR/EMR? (Choose one option)

☐ A clinician or a staff person in the practice

☐ A consultant / service on retainer to the practice

☐ An IT service provider within the health system or organization

☐ Other (please specify): ____________________

____

52. Please indicate if your practice has produced quality reports on any of the following clinical quality measures in the last 6 months. These reports could have been produced by someone on site (i.e., in your practice) or with the assistance of an external group or organization:

a. Percentage of patients aged 18 years and older with Ischemic Vascular Disease (IVD) with documented use of aspirin or other antithrombotic (NQF 0068).

☐ Yes

☐ No

b. Percentage of patients aged 18 through 85 years of age who had a diagnosis of hypertension (HTN) and whose blood pressure (BP) was adequately controlled (<140/90) during the measurement year (NQF 0018).

☐ Yes

☐ No

c. Percentage of patients aged 18 years or older who were screened about tobacco use one or more times within 24 months AND who received cessation counseling intervention if identified as a tobacco user (NQF 0028).

☐ Yes

☐ No

53. Can your practice (or larger organization) report the above quality measures at the practice level?

☐ Yes

☐ No

54. Can your practice (or larger organization) report the above quality measures by clinician (MD, DO, NP, PA)?

☐ Yes

☐ No

55. Does your EHR/EMR vendor help extract data and clinical quality measures?

☐ Yes, and there are restrictions (e.g., a contract saying they own data that prevent data sharing or customizing reports)

☐ Yes, and there are no restrictions

☐ No

☐ Not applicable

☐ Don’t know

56. Overall, how satisfied or dissatisfied are you with your EHR/EMR system?

☐ Very satisfied

☐ Somewhat satisfied

☐ Somewhat dissatisfied

☐ Very dissatisfied

57. At your practice are there plans for installing a new EHR/EMR system within the next 18 months?

☐ Yes

☐ No

☐ Maybe

☐ Unknown

Please continue to SECTION 3 on the next page.

SECTION 3: Improving Patient Care

We would like to learn about the strategies that your practice uses to improve cardiovascular preventive care (e.g., prescribing aspirin for patients at risk for ischemic vascular disease, providing tobacco cessation services for smokers, appropriately managing hypertension, and prescribing statins for high risk patients).

These questions should be completed by one senior member of the practice who has good insights into the clinical operations of the practice, such as a lead clinician or an office manager.

Please continue to Question 58 on the next page.

SECTION 3 Improving Patient Care

58. Indicate the extent to which you agree or disagree that your practice has used the following <u>strategies to improve cardiovascular preventive care</u>:

		STRONGLY DISAGREE	SOMEWHAT DISAGREE	NEITHER AGREE OR DISAGREE	SOMEWHAT AGREE	STRONGLY AGREE	NA
a.	Providing information and skills-training	☐	☐	☐	☐	☐	☐
b.	Using opinion leaders, role modeling, or other vehicles to encourage support for changes	☐	☐	☐	☐	☐	☐
c.	Changing or creating systems in the practice that make it easier to provide high quality care	☐	☐	☐	☐	☐	☐
d.	Removal or reduction of barriers to better quality of care	☐	☐	☐	☐	☐	☐
e.	Using teams focused on accomplishing the change process for improved care	☐	☐	☐	☐	☐	☐
f.	Delegating to non-clinician staff the responsibility to carry out aspects of care that are normally the responsibility of physicians	☐	☐	☐	☐	☐	☐
g.	Providing to those who are charged with implementing improved care the power to authorize and make the desired changes	☐	☐	☐	☐	☐	☐
h.	Periodic measurement of care quality for assessing compliance with any new approach to care	☐	☐	☐	☐	☐	☐
i.	Reporting measurements of practice performance on cardiovascular disease prevention measures (such as aspirin for patients at risk for ischemic vascular disease) for comparison with their peers	☐	☐	☐	☐	☐	☐
j.	Setting goals and benchmarking rates of performance quality on cardiovascular disease prevention measures at least yearly	☐	☐	☐	☐	☐	☐
k.	Customizing the implementation of cardiovascular disease prevention care changes to the practice	☐	☐	☐	☐	☐	☐
l.	Using rapid cycling, piloting, pre-testing, or other vehicles for reducing the risk of negative results for introducing organization-wide change in care	☐	☐	☐	☐	☐	☐
m.	Deliberately designing care improvements so as to make clinician participation less work than before	☐	☐	☐	☐	☐	☐
n.	Deliberately designing care improvements to make the care process more beneficial to the patient	☐	☐	☐	☐	☐	☐

59. Consider all of the priorities your practice has over the next year.
On a scale from 1 to 10 where one is no priority at all and 10 is the highest priority, what is the priority that your practice's leadership places on improving cardiovascular disease preventive care?

1	2	3	4	5	6	7	8	9	10

No priority → **Highest** priority

Thank you for taking the time to complete this survey.
