# Supplementary material for: Disseminating, implementing, and evaluating patient-centered outcomes to improve cardiovascular care using a stepped-wedge design: healthy hearts for Oklahoma
Source: BMC Health Serv Res. 2018 Jun 4;18:404. doi: 10.1186/s12913-018-3189-4 (PMC5987433; doi:10.1186/s12913-018-3189-4)
Supplement: Supplementary file 2 — H2O Practice Member Survey. (PDF 784 kb) [file 12913_2018_3189_MOESM2_ESM.pdf]

P.I. : F. Daniel Duffy, MD
Version: V1.1

OKLAHOMA Clinical and Translational Science Institute
Located at The University of Oklahoma Health Sciences Center

James W. Mold OPHIC
Oklahoma Primary Healthcare Improvement Cooperative

H2O Practice Member Survey

The purpose of this survey is to understand your perceptions of your work environment. No one will see any of your answers linked to your name. All responses to this survey will be reported only as summary results. Please answer each question by checking one box, like this ☑ or this ☒. If you are unsure about how to answer a question, please give the best answer you can.

1. **Practice name:** ____________________
2. **Member ID:** ____________________
3. **Practice Zip code:** ____________________
4. **Date: (mm/dd/yyyy)** ______/______/_________

Please rate your level of agreement with the following statements about your practice. (select only one response per question)

		Strongly Disagree(1)	Disagree (2)	Neutral(3)	Agree(4)	Strongly Agree(5)
5.	Mistakes have led to positive changes here	☐	☐	☐	☐	☐
6.	I have many opportunities to grow in my work	☐	☐	☐	☐	☐
7.	People in our practice actively seek new ways to improve how we do things	☐	☐	☐	☐	☐
8.	People at all levels of this office openly talk about what is and isn't working	☐	☐	☐	☐	☐
9.	Leadership strongly supports practice change efforts	☐	☐	☐	☐	☐
10.	After trying something new, we take time to think about how it worked	☐	☐	☐	☐	☐
11.	Most of the people who work in our practice seem to enjoy their work	☐	☐	☐	☐	☐
12.	It is hard to get things to change in our practice	☐	☐	☐	☐	☐
13.	This practice is a place of joy and hope	☐	☐	☐	☐	☐
14.	This practice learns from its mistakes	☐	☐	☐	☐	☐
15.	Practice leadership promotes an environment that is an enjoyable place to work	☐	☐	☐	☐	☐
16.	People in this practice operate as a real team	☐	☐	☐	☐	☐

Human Research Participant Protection The University of Oklahoma
IRB APPROVED
AAHRPP
IRB NUMBER: 5251
IRB APPROVAL DATE: 10/26/2015

		Strongly Disagree(1)	Disagree (2)	Neutral(3)	Agree(4)	Strongly Agree(5)
17.	When we experience a problem in the practice, we make a serious effort to figure out what's really going on	☐	☐	☐	☐	☐
18.	Leadership in this practice creates an environment where things can be accomplished	☐	☐	☐	☐	☐
19.	We regularly take time to reflect on how we do things	☐	☐	☐	☐	☐
20.	Most people in this practice are willing to change how they do things in response to feedback from others	☐	☐	☐	☐	☐
21.	I can rely on the other people in this practice to do their jobs well	☐	☐	☐	☐	☐
22.	Difficult problems are solved through face-to-face discussions in this practice	☐	☐	☐	☐	☐
23.	Members of this practice are able to bring up problems and tough issues	☐	☐	☐	☐	☐

EvidenceNOW Healthy Hearts for Oklahoma (H2O) is a project designed to help small primary care practices incorporate very recent evidence on how best to deliver cardiovascular disease prevention into their patients' care, including what is known as the ABCS: Aspirin use by high-risk individuals, Blood pressure control, Cholesterol management, and Smoking cessation.

To help with this change process, *EvidenceNOW H2O* will help your practice by offering the assistance of a practice facilitator, external consultants, peer support, and provide opportunities to participate in learning collaboratives. In addition, we will work with you to determine how best to monitor your performance on the ABCS measures.

Please think carefully about the changes we described above.

Please select the response that best reflects your practice's readiness to engage in a change process that involves implementing the changes described above.

		Strongly Disagree(1)	Disagree (2)	Neutral(3)	Agree(4)	Strongly Agree(5)
24.	Our practice is ready to implement these changes	☐	☐	☐	☐	☐

The next set of questions help us understand your perceptions of how your practice addresses patients' needs. Please indicate your level of agreement with the following statements. (select only one response)

		Strongly Disagree(1)	Disagree (2)	Neutral(3)	Agree(4)	Strongly Agree(5)
25.	Our practice does a good job of assessing patient needs and expectations	☐	☐	☐	☐	☐
26.	Our practice uses data from patients to improve care	☐	☐	☐	☐	☐
27.	Our practice uses data on patient expectations and/or experience when developing new services	☐	☐	☐	☐	☐

Using your own definition of <u>burnout</u>, please indicate which of the following statements best describes how you feel about your situation at work. (select only one response)

28.
- ☐1 I enjoy my work. I have no symptoms of burnout
- ☐2 Occasionally I am under stress, and I don't always have as much energy as I once did, but I don't feel burned out
- ☐3 I am definitely burning out and have one or more symptoms of burnout, such as physical and emotional exhaustion
- ☐4 The symptoms of burnout that I'm experiencing won't go away. I think about frustrations at work a lot
- ☐5 I feel completely burned out and often wonder if I can go on practicing. I am at the point where I may need some changes

29. **Are you a clinician (MD, DO, NP, or PA)?**

No.....☐0 ➔ If No, **<u>SKIP</u>** to question **<u>36</u>** (on page **6**)

Yes...☐1 If, Yes, continue with question **30**, below.

⬇

Human Research Participant Protection
The University of Oklahoma
IRB APPROVED
AAHRPP
IRB NUMBER: 5251
IRB APPROVAL DATE: 10/26/2015

NEW JNC8 HYPERTENSION TREATMENT GUIDELINES

In 2014, the JNC8 released new guidelines for treating patients with hypertension that included the following <u>two major changes</u>:

1. BP target for adults over age 60 was changed from <140/90 mmHg to <150/90 mmHg
2. Initial antihypertensive treatment should NOT include an ACE or ARB for black patients

In the next set of questions, please rate your level of agreement regarding whether these new guidelines will lead to better outcomes for your hypertensive patients.

30. **BP target < 150/90 mmHg for patients over 60 years of age**

☐1 This new guideline will improve outcomes for all of my patients
☐2 This new guideline will improve outcomes for some of my patients
☐3 This new guideline will have no impact on outcomes for my patients
☐4 This new guideline will have negative consequences for some of my patients
☐5 This new guideline will have negative consequences for all of my patients

31. **Initial antihypertensive treatment should NOT include an ACE or ARB for black patients**

☐1 This new guideline will improve outcomes for all of my patients
☐2 This new guideline will improve outcomes for some of my patients
☐3 This new guideline will have no impact on outcomes for my patients
☐4 This new guideline will have negative consequences for some of my patients
☐5 This new guideline will have negative consequences for all of my patients

New ACC/AHA Statin Therapy Guidelines

In 2013, the American College of Cardiology and the American Heart Association released new guidelines for treating blood cholesterol to reduce atherosclerotic cardiovascular risk in adults. This included changes to prescribing statins. Statin therapy is now recommended for patients who meet one of the following criteria:

1. Diagnosed with clinical arteriosclerotic cardiovascular disease (ASCVD)
2. Primary elevation of LDL ≥190mg/dL
3. Diagnosed with DM, between 40-75 years old, with LDL between 70-189 mg/dL, and no diagnosis of ASCVD, or
4. No diagnosis of ASCVD or DM, with LDL 70-189 mg/dL and an estimated 10-year ASCVD risk ≥7.5%

Please rate your level of agreement regarding whether these new guidelines will lead to better outcomes for your patients.

32. **Patients diagnosed with ASCVD should be prescribed a statin**

- ☐ 1 This new guideline will improve outcomes for all of my patients
- ☐ 2 This new guideline will improve outcomes for some of my patients
- ☐ 3 This new guideline will have no impact on outcomes for my patients
- ☐ 4 This new guideline will have negative consequences for some of my patients
- ☐ 5 This new guideline will have negative consequences for all of my patients

33. **Patients with a primary elevation of LDL >190mg/dL should be prescribed a statin**

- ☐ 1 This new guideline will improve outcomes for all of my patients
- ☐ 2 This new guideline will improve outcomes for some of my patients
- ☐ 3 This new guideline will have no impact on outcomes for my patients
- ☐ 4 This new guideline will have negative consequences for some of my patients
- ☐ 5 This new guideline will have negative consequences for all of my patients

34. **Patients with a diagnosis of DM, between 40-75 years old, with LDL between 70-189 mg/dL, and no diagnosis of ASCVD should be prescribed a statin**

- ☐ 1 This new guideline will improve outcomes for all of my patients
- ☐ 2 This new guideline will improve outcomes for some of my patients
- ☐ 3 This new guideline will have no impact on outcomes for my patients
- ☐ 4 This new guideline will have negative consequences for some of my patients
- ☐ 5 This new guideline will have negative consequences for all of my patients

35. **Patients with no diagnosis of ASCVD or DM, with LDL 70-189 and an estimated 10-year ASCVD risk ≥7.5% should be prescribed a statin**

- ☐ 1 This new guideline will improve outcomes for all of my patients
- ☐ 2 This new guideline will improve outcomes for some of my patients
- ☐ 3 This new guideline will have no impact on outcomes for my patients
- ☐ 4 This new guideline will have negative consequences for some of my patients
- ☐ 5 This new guideline will have negative consequences for all of my patients

Human Research Participant Protection
The University of Oklahoma
IRB APPROVED
AAHRPP
IRB NUMBER: 5251
IRB APPROVAL DATE: 10/26/2015

Please complete the following information about yourself.

36. **What is your role in this practice?** (select only one option)

- ☐ 1 Physician (MD, DO)
- ☐ 2 Nurse Practitioner or Physician Assistant
- ☐ 3 Clinical staff (e.g., RN, LPN, MA, CMA, Behavioral health providers)
- ☐ 4 Non-clinical staff (e.g., receptionist, billing staff)
- ☐ 5 Office manager
- ☐ 6 Other

↳ 36a. **If "Other" role was checked, please specify:** ________________________________

37. **How many years have you worked in this practice?**
Please round to the nearest year. If you have worked in the practice less than one year, please indicate that by checking that response option.

_______ **years** **OR** ☐ **I have worked in this practice less than one year.**

38. **How many hours per week do you work at this practice?**

_______ **hours/week**

Thank you for taking the time to complete this survey.

Human Research Participant Protection
The University of Oklahoma
IRB APPROVED
AAHRPP
IRB NUMBER: 5251
IRB APPROVAL DATE: 10/26/2015
